# Deep Learning-Based Radiomics of B-Mode Ultrasonography and Shear-Wave Elastography: Improved Performance in Breast Mass Classification

**DOI:** 10.3389/fonc.2020.01621

**Published:** 2020-08-28

**Authors:** Xiang Zhang, Ming Liang, Zehong Yang, Chushan Zheng, Jiayi Wu, Bing Ou, Haojiang Li, Xiaoyan Wu, Baoming Luo, Jun Shen

**Affiliations:** ^1^Department of Radiology, Sun Yat-sen Memorial Hospital, Sun Yat-sen University, Guangzhou, China; ^2^Guangdong Provincial Key Laboratory of Malignant Tumor Epigenetics and Gene Regulation, Medical Research Center, Sun Yat-sen Memorial Hospital, Sun Yat-sen University, Guangzhou, China; ^3^Department of Ultrasound, Sun Yat-sen Memorial Hospital, Sun Yat-sen University, Guangzhou, China; ^4^Department of Radiology, Sun Yat-sen University Cancer Center, Sun Yat-sen University, Guangzhou, China

**Keywords:** deep learning, radiomics, ultrasonography, shear-wave elastography, breast neoplasms

## Abstract

**Objective:**

Shear-wave elastography (SWE) can improve the diagnostic specificity of the B-model ultrasonography (US) in breast cancer. However, whether deep learning-based radiomics signatures based on the B-mode US (B-US-RS) or SWE (SWE-RS) could further improve the diagnostic performance remains to be investigated. We aimed to develop the B-US-RS and SWE-RS and determine their performances in classifying breast masses.

**Materials and Methods:**

This retrospective study included 291 women (mean age ± standard deviation, 40.9 ± 12.3 years) from two centers who had US-visible solid breast masses and underwent biopsy and/or surgical resection between June 2015 and July 2017. B-mode US and SWE images of the 198 masses in 198 patients (training cohort) from center 1 were segmented, respectively, to construct B-US-RS and SWE-RS using the least absolute shrinkage and selection operator regression and tested in an independent validation cohort of 65 masses in 65 patients from center 1 and in an external validation cohort of 28 masses in 28 patients from center 2. The performances of B-US-RS and SWE-RS were assessed using receiver operating characteristic (ROC) analysis and compared with that of radiologist assessment [Breast Imaging Reporting and Data System (BI-RADS)] and quantitative SWE parameters [maximum elasticity (*E*_max_), mean elasticity (*E*_mean_), elasticity ratio (*E*_ratio_), and elastic modulus standard deviation (*E*_SD_)] by using the McNemar test.

**Results:**

The single best-performing quantitative SWE parameter, *E*_max_, had a higher specificity than BI-RADS assessment in the training and independent validation cohorts (*P* < 0.001 for both). The areas under the ROC curves (AUCs) of B-US-RS and SWE-RS both were 0.99 (95% CI = 0.99–1.00) in the training cohort, 1.00 (95% CI = 1.00–1.00) in the independent validation cohort, and 1.00 (95% CI = 1.00–1.00) in the external validation cohort. The specificities of B-US-RS and SWE-RS were higher than that of *E*_max_ in the training (*P* < 0.001 for both) and independent validation cohorts (*P* = 0.02 for both).

**Conclusion:**

The B-US-RS and SWE-RS outperformed the quantitative SWE parameters and BI-RADS assessment for classifying breast masses. The integration of the deep learning-based radiomics approach would help improve the classification ability of B-mode US and SWE for breast masses.

## Introduction

Breast ultrasonography (US), avoiding the ionizing radiation and the requirement for breast compression, is a valuable supplemental screening tool in women, in particular those with dense breasts and negative mammogram results (1, 2). The Breast Imaging Reporting and Data System (BI-RADS) provides a standardized terminology to make an assessment and subsequent recommendation for lesions detected by US (3). For a lesion with BI-RADS category 3 (probably benign, ≤2% likelihood of malignancy), a short-interval follow-up was recommended instant of immediate biopsy (4). In contrast, for a lesion with BI-RADS category 4a (low suspicion of malignancy, >2%, but ≤10% likelihood of malignancy) or higher, further biopsy would be recommended (4). Improved classification of breast lesions might allow some benign lesions to be downgraded from BI-RADS category 4a to 3, where surveillance with safe follow-up would be an alternative to biopsy. US is very sensitive for breast lesion detection. However, the low specificity (high false-negative) in the differentiation of benign from malignant breast masses remains a major limitation of B-mode US (2, 5), which might lead to more benign lesions undergoing unnecessary biopsy. Elastographic US, including strain and shear-wave elastography (SWE), both of which are based on tissue stiffness, has the potential to improve the diagnostic specificity of B-mode breast US (6, 7). Strain elastography is based on the relative displacement of the tissue by freehand external compression. It has the shortcoming of being operator-dependent, and substantial varying degrees of interobserver variability may occur during data acquisition and interpretation on some vendors (8). Although the semiquantitative parameters (i.e., elastographic-to-B-mode length ratio and strain ratio) for strain are available (9), the exact elasticity value cannot be quantified (10–12). SWE can provide quantitative elasticity parameters and display a visual color overlay of elastic information during real-time imaging *via* the usage of acoustic radiation force induced by the fixed ultrasound push pulse generated from the transducer (10, 11). Several studies have shown that quantitative SWE parameters are reproducible for assessing elastographic features of breast masses and can improve the diagnostic specificity of B-mode US without loss of sensitivity (5, 7, 13). However, the specificity remains limited up to 86% when the quantitative SWE parameters were used (14). Therefore, a method to improve the diagnostic performance, especially to further improve the specificity of B-mode US or SWE, for the classification of breast lesions is needed.

Radiomics can extract high-throughput quantitative data from the medical image and objectively evaluate the inter- and intra-neoplastic heterogeneity through the spatial distribution of voxel intensity, which cannot be directly detected by the unaided eye (15, 16). Deep learning radiomics is one of the methods which can extract a large number of quantitative features from radiologic images by supervised learning (16). It is different from the traditional radiomics method in that, instead of extracting features in a hand-designed approach, deep learning only needs minor preprocessing of the data, if necessary, and then extracts informative representations in a self-learning manner (17). Although deep learning-based radiomic features are difficult to interpret, deep learning techniques have shown promising capabilities for the extraction of correlative quantitative representation in several medical applications (17, 18). Recently, deep learning based on the convolutional neural network has been considered as a stable, effective approach for the feature extraction, classification, detection, and segmentation tasks of radiologic images (17–20). It has been shown that a deep learning-based radiomics signature based on US and SWE could serve as a reliable and powerful tool for the prediction of axillary lymph node status in early-stage breast cancer (21). However, whether a deep learning-based radiomics signature can be used to improve the diagnostic performance of B-mode US and SWE for the classification of breast lesions remains unknown.

We hypothesized that deep learning-based radiomics signatures derived from B-mode US images (B-US-RS) and SWE images (SWE-RS) have better diagnostic performance than those of quantitative SWE parameters and radiologist assessment in classifying breast masses. The purpose of this study was to develop B-US-RS and SWE-RS and determine their diagnostic performances in classifying breast masses as compared with quantitative SWE parameters and radiologist assessment.

## Materials and Methods

### Patients and Lesions

**FIGURE 1:**
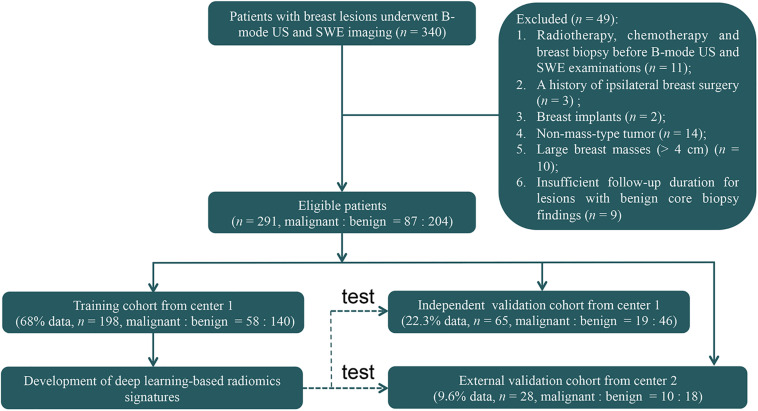
The flowchart shows the enrollment pathway in this study and the distribution of patients in the training and validation cohorts. *US*, ultrasonography; *SWE*, shear-wave elastography.

This retrospective study was reviewed and approved by the ethics committee of center 1 (Sun Yat-sen Memorial Hospital, Sun Yat-sen University, Guangzhou, China) and center 2 (Guangdong Provincial Traditional Chinese Medicine Hospital, Guangzhou, China). Patient informed consent was waived because of the retrospective nature of this study. Between June 2015 and July 2017, 340 consecutive women who underwent breast B-mode US and SWE examinations and had US-visible solid breast masses were identified (Figure 1). The inclusion criteria were women who had US-visible solid breast masses and who underwent biopsy and/or surgical resection. The exclusion criteria were as follows: (1) radiotherapy, chemotherapy, or breast biopsy before B-mode US and SWE examinations; (2) a history of ipsilateral breast surgery; (3) breast implant; (4) non-mass-type lesion; (5) large breast masses (>4 cm) beyond the maximum range of SWE detection; and (6) insufficient follow-up duration (<2 years of follow-up for lesions with benign core biopsy findings).

Finally, 263 women (mean age = 40.9 ± 12.3 years, range = 18–77 years) with 263 breast masses (mean size = 1.3 ± 0.6 cm, range = 0.4–4.2 cm) from center 1 and 28 women (mean age = 40.8 ± 12.1 years, range = 24–68 years) with 28 breast masses (mean size = 1.3 ± 0.6 cm, range = 0.5–3.4 cm) from center 2 were included for analysis. These 263 patients from center 1 were divided 3:1 into the training cohort and independent validation cohort. Among them, 198 patients (mean age = 40.7 ± 12.1 years, range = 18–77 years) with 198 masses (mean size = 1.3 ± 0.6 cm, range = 0.4–3.4 cm) between June 2015 and December 2016 were identified to comprise the training cohort, which was used for radiomics signature construction, and 65 patients (mean age = 41.5 ± 13.2 years, range = 19–70 years) with 65 masses (mean size = 1.3 ± 0.6 cm, range = 0.4–3.4 cm) between January 2017 and May 2017 were identified as an independent validation cohort. Then, 28 patients with 28 masses between January 2017 and July 2017 from center 2 were identified as an external validation cohort. The 65 lesions in the independent validation cohort and the 28 masses in the external validation cohort were not used for radiomics signature development. There were no significant differences in the age (*P* = 0.90) and mass size (*P* = 0.96) between the training and the two validation cohorts.

In the training and validation cohorts, all masses were pathologically confirmed through US-guided core needle biopsy after breast B-mode US and SWE examination. The mass was resected in any malignant, atypical, or high-risk core biopsy result (i.e., lobular carcinoma *in situ*, atypical ductal hyperplasia, radial scar, and papillary lesion) and the diagnosis was confirmed by surgical pathology. Surgical excision was performed for 102 masses (51.5%) in the training cohort, 21 masses (32.3%) in the independent validation cohort, and 14 masses (50%) in the external validation cohort. For benign masses not treated by surgical resection, the diagnosis was further confirmed by follow-up US. The mean duration of follow-up with the US was 31 months (range = 24–42 months), and lesion stability was confirmed in all patients.

### B-Mode US and SWE Acquisition

The B-mode US and SWE acquisition were performed by one of the two radiologists (BO and ML) in center 1 and one radiologist (Shulian Zhuang) in center 2 by using the US system (Aixplorer, SuperSonic Imagine, Aix-en-Provence, France) equipped with a multifrequency linear transducer (SL15–4, SuperSonic Imagine, Aix-en-Provence, France) operating at 4–15 MHz, according to the American Institute of Ultrasound in Medicine practice guidelines (3). The three radiologists had 15, 5, and 5 years of breast US experience, respectively, and at least 4 years (at least 150 patients per year) of experience of breast SWE. Clinical and mammographic findings (if any) of patients were available before B-mode US and SWE acquisition. After the B-mode US, the SWE image was acquired at a plane that showed the largest diameter of the breast mass. During SWE image acquisition, the scanning pressure applied by the operator was as low as possible to reduce artifactual stiffness, and the probe was kept still with no pressure being applied to the mass for a few seconds until the stable image was build up; meanwhile, patients were asked to hold their breath. A rectangular region of interest (ROI) was set for SWE acquisition. The size and location of the ROI were standardized, as previously reported (22). The stiffness in the ROI was displayed as a color map. This color-coded map represents quantitative values for the Young elastic modulus (in kilopascals) at each pixel, on which very soft tissues were coded in dark blue and areas of increasing stiffness were coded in light blue, green, orange, and red (22).

### Radiologist Assessment and SWE Quantitative Analysis

In center 1, the radiologist assessment of the BI-RADS categories was recorded by one of two radiologists (B.O. or M.L.) after B-mode US imaging acquisition according to the American Institute of Ultrasound in Medicine practice guidelines (3). The other radiologist reviewed the assessment result, and in the case of a disagreement, a consensus was reached. In center 2, the radiologist assessment of the BI-RADS categories was recorded by the radiologist (S.L.Z.). The expected malignancy rates of the BI-RADS categories (23) are as follows: category 3 (probably benign, ≤2% likelihood of malignancy); category 4a (low suspicion of malignancy, >2%, but ≤10% likelihood of malignancy); category 4b (intermediate suspicion of malignancy, >10%, but ≤50% likelihood of malignancy); category 4c (moderate suspicion of malignancy, >50%, but <95% likelihood of malignancy); and category 5 (highly suggestive of malignancy, ≥95% likelihood of malignancy).

**FIGURE 2:**
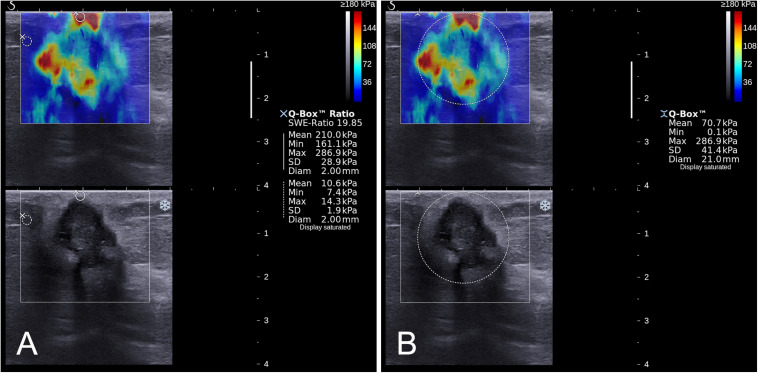
Images show a grade 3 invasive ductal carcinoma in a 67-year-old woman. A standardized rectangular region of interest (ROI) was set for shear-wave elastography (SWE) image acquisition, and stiffness was displayed as a *color map* in the rectangular ROI **(A,B)**. Quantitative SWE parameters including *E*_max_ (286.9 kPa), *E*_mean_ (210.0 kPa), and *E*_ratio_ (19.85) were measured by using two 2-mm^2^ round ROIs. One was placed within or adjacent to the mass to encompass the maximum stiffness area, and the other one placed at the normal fatty tissue outside the lesion, but within the square ROI **(A)**. Another round ROI adjusted to the mass contour to encompass the maximum area of mass was used to measure *E*_SD_ (41.4 kPa; **(B)**).

The quantitative SWE parameters were independently measured by one of the radiologists (B.O. or M.L.) in center 1 and the radiologist (S.L.Z.) in center 2 who had performed the B-mode US and SWE imaging. Quantitative SWE parameters were measured by using two 2-mm^2^ round ROIs. The method of ROI placement is shown in Figure 2. One round ROI was placed within or adjacent to the mass to encompass the maximum stiffness area, but not including the tissue outside the lesion displayed on the B-mode image, and the other round ROI was placed at the normal fatty tissue outside the lesion, but within the rectangular ROI which was set for SWE acquisition (7, 13). Quantitative SWE parameters, including maximum elasticity (*E*_max_), mean elasticity (*E*_mean_), and elasticity ratio (*E*_ratio_), were automatically calculated and visualized by the US system. *E*_ratio_ is the ratio of the *E*_mean_ in the maximum stiffness area of the mass to the *E*_mean_ in the ROI in the normal fatty tissue outside the lesion. Then, a round ROI adjusted to the mass contour to encompass the maximum area of mass was placed on the B-mode image, and the elastic modulus standard deviation (*E*_SD_) was automatically calculated. For each patient, these four quantitative SWE parameters were measured three times, and the maximum of *E*_max_ and the median of *E*_mean_, *E*_ratio_, and *E*_SD_ were selected for analysis. The same view of B-mode US and SWE images displaying the maximal diameter of the lesion was used for further imaging segmentation. Quantitative SWE parameters were not used for the assessment of the BI-RADS category.

### Lesion Segmentation

**FIGURE 3:**
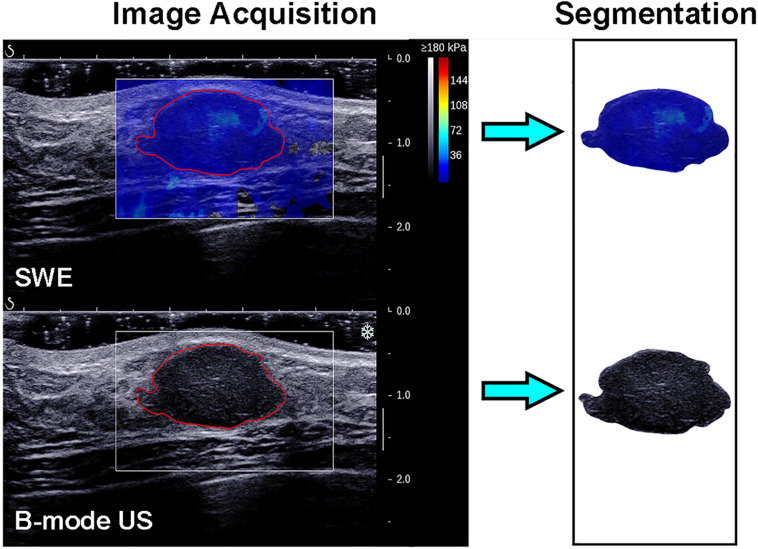
Images show a fibroadenoma in a 41-year-old woman. *Top left*: The shear-wave elastography (SWE) image shows a homogeneous mass, the region of interest encompassing the whole mass, and the contour line located in the border of the mass. *Bottom left*: B-mode ultrasonography (US) image shows an irregular hypoechoic mass considered to be a Breast Imaging Reporting and Data System category 3 lesion, and the region of interest encompassed the hypoechoic region which represented the tumor. The segmented SWE image (*top right in black box*) and B-mode US image (*bottom right in black box*) were used for further deep learning-based radiomic feature extraction.

**FIGURE 4:**
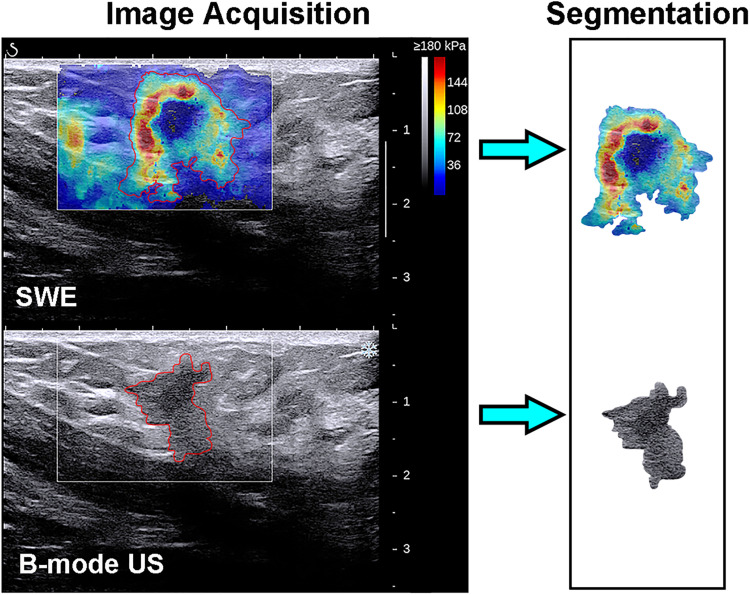
Images show a grade 3 invasive ductal carcinoma in a 58-year-old woman. *Top left*: The shear-wave elastography (SWE) image shows a non-homogeneous mass, the region of interest encompassing the whole mass and adjacent breast tissue, and the contour line located in the border of *light blue* and *green*. *Bottom left*: B-mode ultrasonography (US) image shows an irregular hypoechoic mass considered to be a Breast Imaging Reporting and Data System category 4c lesion, and the region of interest encompassed the hypoechoic region which represented the tumor. The segmented SWE image (*top right in black box*) and B-mode US image (*bottom right in black box*) were used for further deep learning-based radiomic feature extraction.

The recorded B-mode US and SWE images were manually segmented using an open-source imaging platform (ITK-SNAP, version 3.6.0; www.itksnap.org) by one investigator (investigator 1: M.L., with 5 years of experience in breast US, and 4 years of experience in breast SWE) who was blinded to the pathologic results of breast lesions. For the segmentation of the B-mode US images, a two-dimensional ROI was drawn on the B-mode grayscale US image, encompassing the hypoechoic region, which represents the mass. For the segmentation of the SWE image, the B-mode US image was used as a reference, and a two-dimensional ROI was drawn on the SWE color-coded image within the regions of square ROI embedded. Homogeneous masses (often soft masses and dark blue or light blue on SWE) are less likely to be malignant lesions, while non-homogeneous masses (often stiff masses and orange or red on SWE) are more likely to be malignant lesions on SWE images (7). Thus, the ROI encompassed the whole mass, and the contour line was placed along the border of the mass on the B-mode US image for homogeneous masses (Figure 3). The ROI covered the whole mass and adjacent breast tissue for non-homogeneous masses (Figure 4) as the maximum area of stiffness in malignant lesions is always found in the peritumoral region rather than in the lesion itself (24).

Among the 198 masses in the training cohort, 50 masses were randomly selected, and the same segmentation procedure was repeated by the other investigator (investigator 2: JW, with 8 years of experience in breast US and 3 years of experience in breast SWE imaging) who was blinded to the pathologic result and then repeated by investigator 1 one month later. The intra- and inter-rater reproducibility of breast lesion segmentation was performed by using the Dice similarity coefficient (25). The intra- and inter-rater reproducibility of deep learning-based radiomic feature extraction was also assessed, and the intra- and interclass correlation coefficients (ICC) were calculated. A Dice similarity coefficient ranging from 0.75 to 1.00 was defined as an excellent agreement, from 0.50 to 0.74 as a good agreement, from 0.25 to 0.49 as a moderate agreement, and less than 0.25 as a poor agreement (26). An ICC ranging from 0.81 to 1.00 was defined as an almost perfect agreement, from 0.61 to 0.80 as a substantial agreement, from 0.41 to 0.60 as a moderate agreement, from 0.21 to 0.40 as a fair agreement, and from 0 to 0.20 as weak or no agreement (27). An ICC greater than 0.6 is considered a satisfactory agreement for deep learning-based radiomic feature extraction (28).

### Radiomic Feature Extraction

**FIGURE 5:**
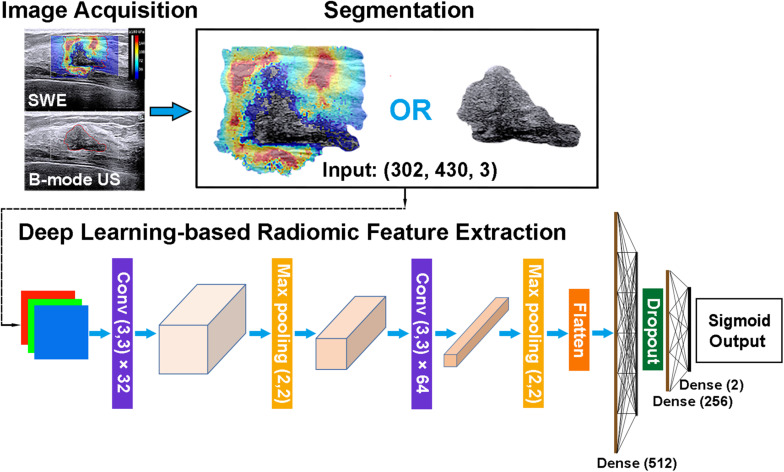
Major steps for the image acquisition, segmentation, and deep learning-based radiomic feature extraction method. *SWE*, shear-wave elastography; *US*, ultrasonography; and *Conv*, convolutional layer.

Radiomic features can be extracted through deep learning approaches (29). The extracted deep learning-based radiomic features could be adaptively learned from images and better correlated with the specific image datasets. Thus, the deep learning-based radiomic features of masses were, respectively, extracted from B-mode US and SWE images by using an open-source platform (Tensorflow, version 1.7.0; https://www.tensorflow.org). To extract deep learning-based radiomic features, a convolutional neural network, which mainly contains two blocks of convolution and pool layers followed by three fully connected layers, was used (Figure 5). The bounding box of ROIs was, respectively, extracted from the segmented B-mode US and SWE images and resized to the dimension of 430 × 302 as the input. Two convolutional layers with a kernel size of 3 × 3 and depths of 32 and 64 were utilized, and the “rectified linear unit (ReLU)” operator was used as the activation function. Each convolutional layer was followed by a max pooling layer (kernel size, 2 × 2) to refine the features and reduce computational complexity. After the second convolutional layer, the flattened feature map was connected with a fully connected layer with nodes of 512, a dropout layer with the rate of 0.5, and two more fully connected layers with nodes of 256 and 2, respectively. The diagnosis of each case as benign or malignant was used as the training label. To train and fine-tune the network, the original training set was further divided into a training and an internal validation set at the ratio of 8:2. On-the-fly data augmentations such as horizontal and vertical image flipping and rescaling were performed during the training to improve training data variety. The network was trained by 80 epochs using a stochastic gradient descent optimizer with a learning rate of 0.01 and category cross-entropy as the loss function. The best model was selected based on performance evaluated on the internal validation set. After the network is well trained, the output feature maps of the second and third to the last fully connected layers were defined as the deep learning-based radiomic features, which have a dimension of 768. The source code and the trained model supporting the finding of this study are available at https://github.com/biototem/ultrasound_image_classification.

### Feature Selection, Development, and Validation of Radiomics Signature

A three-step procedure was performed for dimensionality reduction, robust deep learning-based radiomic feature selection, and radiomics signature construction. Firstly, the deep learning-based radiomic features with both intra-rater (reader 1 for twice assessment) and inter-rater (reader 1 and reader 2) ICC > 0.6 were selected (28). Secondly, the deep learning-based radiomic features were reduced by using the least absolute shrinkage and selection operator (LASSO) regression method, which is available for the regression of high-dimensional data (30). The LASSO regression method was used to select the breast mass classification-related features with non-zero coefficients from the training cohort. Lastly, the radiomics score (rad-score) was computed for each patient through the LASSO regression with a combination of selected features weighted by their respective coefficients. Both feature selection and the subsequent radiomics signature development were performed in the training cohort. The performance of the obtained radiomics signature was, respectively, evaluated using an independent validation cohort and an external validation cohort, which was not used for model development.

### Statistical Analysis

All numerical data were presented as the mean ± standard deviation, unless otherwise indicated. Continuous variables, including the age and maximum diameter of the lesion between the benign and malignant groups in the training and validation cohorts, were compared by using Student’s *t* test or Mann–Whitney *U* test, when appropriate. Categorical variables, including clinical symptoms, side of the lesion, and family history of breast cancer, were compared by using the χ^2^ test. LASSO regression was used to select the deep learning-based radiomic features by using the in-house package, including “matrix,” “foreach,” “pROC,” and “glmnet,” of R software (version 3.0.1; R Foundation for Statistical Computing, 2013). The sensitivity, specificity, and likelihood ratio were calculated, and the Youden index was used to determine the optimal threshold. The sensitivity and specificity of the SWE-RS, B-US-RS, quantitative SWE parameters, and the BI-RADS category were compared by using the McNemar test. The areas under the receiver operating characteristic curves (AUCs) were compared by using the method proposed by DeLong (31). The interpretation of the likelihood ratio was based on the guide proposed by Jaeschke et al. (32), in which likelihood ratios greater than 10 or less than 0.1 generate large and often conclusive changes in the posttest probability, likelihood ratios between 5 and 10 or 0.1 and 0.2 generate moderate shifts in posttest probability, and likelihood ratios less than 5 or greater than 0.2 generate small changes in probability. Statistical analysis was performed using SPSS (version 22.0; IBM, 2013; continuous and categorical variables, ICC, and sensitivity and specificity calculation and comparison) and R software [receiver operating characteristic (ROC), comparison of AUCs, and LASSO regression]. A two-sided *P* value less than 0.05 was considered to indicate statistical significance. The *P* value for statistical significance was corrected by Bonferroni correction when multiple testing was used.

## Results

### Clinicopathologic Characteristics of Breast Lesions

The clinicopathologic characteristics of 291 patients are shown in Table 1. The BI-RADS categories are shown in Table 2. There were 291 masses assessed. Among the 291 masses, 87 (29.9%) were malignant and 204 (70.1%) were benign. The age of the patients with malignant masses was greater than the patients with benign ones in the training and the two validation cohorts (*P* < 0.001 for all). Among all 291 women, 153 (52.6%) had clinical symptoms, including a palpable breast mass (*n* = 142) and nipple discharge (*n* = 11), and the remaining 138 (47.4%) women were asymptomatic. One hundred and thirty-seven (47.1%) women had right breast lesions and 154 (52.9%) women had left breast masses. Overall, the maximum diameter of the malignant masses was larger than that of the benign lesions either in the training cohort (mean size = 1.6 ± 0.7 cm *vs*. 1.2 ± 0.5 cm; *P* < 0.001) or the independent validation cohort (mean size = 1.7 ± 0.7 cm *vs*. 1.2 ± 0.5 cm; *P* = 0.002).

**TABLE 1:** Clinicopathologic characteristics of 291 patients.

	Training cohort (*n* = 198)			Independent validation cohort (*n* = 65)			External validation cohort (*n* = 28)		
			
Characteristic	Malignant (*n* = 58)	Benign (*n* = 140)	*P* value	Malignant (*n* = 19)	Benign (*n* = 46)	*P* value	Malignant (*n* = 10)	Benign (*n* = 18)	*P* value
Age (years)	50.7 ± 11.2	36.6 ± 9.9	<0.001	54.8 ± 8.4	36.0 ± 10.7	<0.001	49.7 ± 10.2	35.9 ± 9.5	<0.001
Clinical symptom			<0.001			0.01			0.004
Palpable breast mass	32	62		11	17		9	11	
Nipple discharge	6	1		2	0		0	2	
Asymptomatic	20	77		6	29		1	5	
Laterality			0.55			0.33			0.74
Left	30	79		7	23		5	10	
Right	28	61		12	23		5	8	
Family history of breast cancer			0.67			0.07			0.40
Yes	11	23		0	7		1	4	
None	47	117		19	39		9	14	
Maximum diameter on B-mode US (cm)	1.6 ± 0.7	1.2 ± 0.5	<0.001	1.7 ± 0.7	1.2 ± 0.5	0.002	1.5 ± 0.8	1.2 ± 0.5	0.26

**TABLE 2:** Histopathologic diagnoses of 291 breast masses.

Histopathologic result	BI-RADS 3 (*n* = 112)		BI-RADS 4a (*n* = 62)		BI-RADS 4b (*n* = 45)		BI-RADS 4c (*n* = 52)		BI-RADS 5 (*n* = 20)	
Histopathologic diagnosis	Benign (*n* = 108)	Malignant (*n* = 4)	Benign (*n* = 59)	Malignant (*n* = 3)	Benign (*n* = 36)	Malignant (*n* = 9)	Benign (*n* = 1)	Malignant (*n* = 51)	Benign (*n* = 0)	Malignant (*n* = 20)
Histopathologic subtypes	Fibroadenoma (*n* = 68); ANDI (*n* = 36); benign phyllodes tumor (*n* = 3); tubular adenoma (*n* = 1)	IDC (*n* = 4)	Fibroadenoma (*n* = 33); ANDI (*n* = 19); IP (*n* = 3); benign phyllodes tumor (*n* = 1); complicated cyst (*n* = 2); scar (*n* = 1)	IDC (*n* = 2), DCIS (*n* = 1)	Fibroadenoma (*n* = 22); ANDI (*n* = 10); IP (*n* = 2); complicated cyst (*n* = 1); benign phyllodes tumor (*n* = 1)	IDC (*n* = 6); DCIS (*n* = 2); IPC (*n* = 1)	Fibroadenoma (*n* = 1)	IDC (*n* = 47); DCIS (*n* = 3); ILC (*n* = 1)	–	DCIS (*n* = 17); ILC (*n* = 2); IPC (*n* = 1)

**ANDI*, aberrations of normal development and involution without fibroadenoma (mainly consisting of a spectrum of fibrocystic and proliferative changes); *IDC*, invasive ductal carcinoma; *IP*, intraductal papilloma; *DCIS*, ductal carcinoma *in situ*; *IPC*, invasive papillary carcinoma; and *ILC*, invasive lobular carcinoma.*

### Feature Selection

**FIGURE 6:**
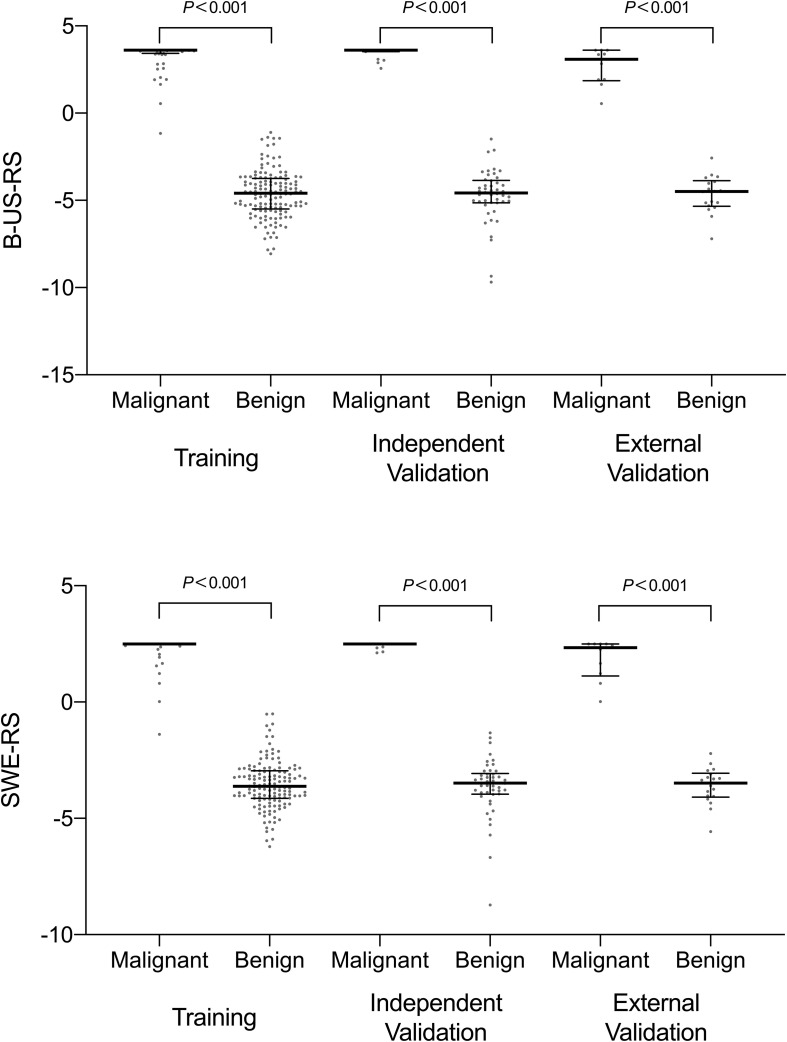
Scatter plots of the radiomics score between benign and malignant lesions in the training, independent validation, and external validation cohorts. The *dots* represent the distribution of the radiomics score; the *dots at the top* and *bottom* represent the maximum and minimum values of the radiomics score, respectively. The *long horizontal line in the middle* represents the median; the *top* and *bottom of the whiskers* represent the 75th and 25th percentiles, respectively. *B-US-RS*, deep learning-based radiomics signature of B-mode ultrasonography; *SWE-RS*, deep learning-based radiomics signature of shear-wave elastography.

The intra-rater Dice similarity coefficient calculated based on reader 1’s twice segmentation ranged from 0.82 to 0.97 on the B-mode US image and from 0.81 to 0.93 on the SWE image, and the inter-rater Dice similarity coefficient calculated based on reader 1’s first-extracted features and those of reader 2 ranged from 0.78 to 0.97 on the B-mode US image and from 0.76 to 0.91 on the SWE image, indicating an excellent intra- and inter-rater consistency for lesion segmentation. The intra-rater ICC ranged from 0.47 to 0.98 on the B-mode US image and from 0.51 to 0.97 on the SWE image, and the inter-rater ICC ranged from 0.41 to 0.95 on the B-mode US image and from 0.61 to 0.91 on the SWE image, indicating a satisfactory intra- and inter-rater reproducibility for deep learning-based radiomic feature extraction. Among the 768 deep learning-based radiomic features from the B-mode US and 768 deep learning-based radiomic features from SWE, 472 features with ICC > 0.6 from B-mode US and 577 features with ICC > 0.6 from SWE were selected, respectively. Between the selected features for reader 1 twice as well as reader 1 and reader 2, no statistically significant difference was found either in the 472 features from B-mode US (*P* values ranged from 0.55 to 0.88) or the 577 features from SWE (*P* values ranged from 0.46 to 0.81). Therefore, further analysis regarding the radiomics signature construction was based on the deep learning-based radiomic features extracted by reader 1. According to the results of the LASSO regression, seven deep learning-based radiomic features of B-mode US and four deep learning-based radiomic features of SWE were, respectively, selected for the development of B-US-RS and SWE-RS. The radiomics signature of B-mode US and SWE was, respectively, constructed, with the rad-score calculated, by using the following formulas: rad-score for B-US = 3.6044336-0.3351454 × US 747-0.5255682 × US 637-0.2029134 × US 535-0.8266571 × US 719-0.7043252 × US 518-0.7884457 × US 565-0.9791398 × US 532. The rad-score for SWE = 2.496014-0.3666784 × SWE 518-1.4319200 × SWE 532-0.4749501 × SWE 565-0.2671713 × SWE 719. The rad-scores between the benign and malignant lesions in the training, independent validation, and external validation cohorts are shown in Figure 6 and Supplementary Table S1.

### Diagnostic Performances of B-US-RS, SWE-RS, Quantitative SWE Parameters, and BI-RADS Assessment in the Training Cohort

**FIGURE 7:**
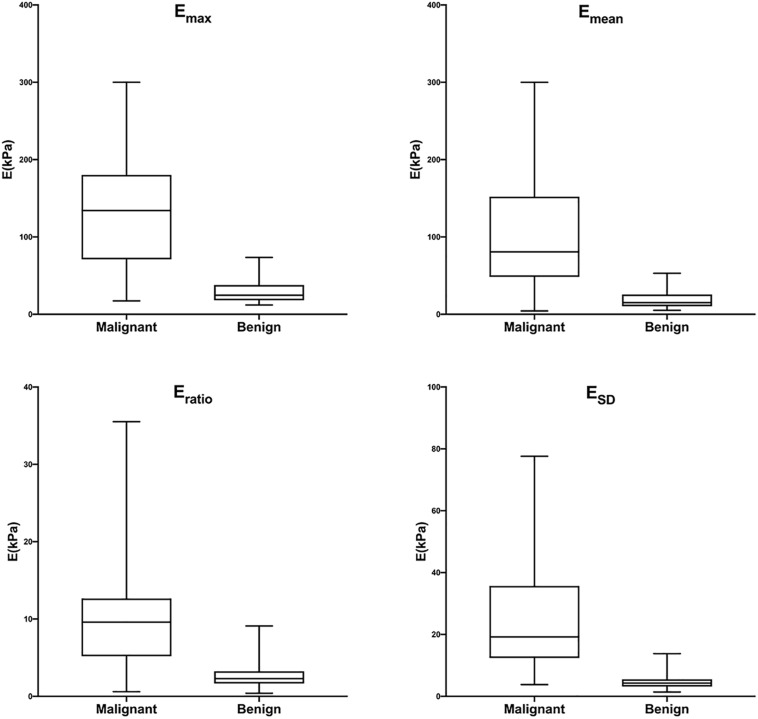
Box-and-whisker plots of *E*_max_, *E*_mean_, *E*_ratio_, and *E*_SD_ in malignant and benign lesions in the training cohort. The *top* and *bottom of each box* represent the 75th and 25th percentiles, respectively. The *horizontal line in each box* represents the median; the *top* and *bottom of the whiskers* represent the minimum and maximum values, respectively. *E*_max_, *E*_mean_, *E*_ratio_, and *E*_SD_ were significantly higher in malignant lesions than in benign lesions (*P* < 0.001 for all).

The training cohort included 198 masses, of which 140 (70.7%) were benign and 58 (29.3%) were malignant. The diagnostic performances of B-US-RS, SWE-RS, quantitative SWE parameters, and BI-RADS assessment in the training cohort are shown in Table 3. The quantitative SWE parameters, including *E*_max_, *E*_mean_, *E*_ratio_, and *E*_SD_, were significantly higher in malignant lesions than in benign lesions (*P* < 0.001 for all; Figure 7). Among these SWE parameters, *E*_max_ achieved the highest AUC [0.92; 95% confidence interval (CI) = 0.88–0.96] and was chosen for further comparative analysis, though there were no significant differences in the AUCs between *E*_max_ and *E*_mean_ (*P* = 0.45) as well as *E*_max_ and *E*_SD_ (*P* = 0.91). Moreover, no significant difference was found in the AUCs between *E*_max_ and the four quantitative SWE parameters combined (0.92; 95% CI = 0.88–0.97, *P* = 0.81). The AUCs were not significantly different between *E*_max_ and BI-RADS assessment (0.94; 95% CI = 0.89–0.98, *P* = 0.36). The specificity of *E*_max_ was higher (*P* < 0.001) while its sensitivity was lower (*P* = 0.04) than that of BI-RADS assessment.

**TABLE 3:** Diagnostic performances of B-US-RS, SWE-RS, quantitative SWE parameters, and BI-RADS assessment in the training cohort.

Parameter	Threshold	Sensitivity (%)*	Specificity (%)*	AUC*	Positive likelihood ratio	Negative likelihood ratio
B-US-RS	−1.28	100 (92–100) [58/58]	99 (96–100) [139/140]	0.99 (0.99–1.00)	140 (19.86–986.92)	0 (0–0)
SWE-RS	−0.24	98 (99–100) [57/58]	100 (97–100) [140/140]	0.99 (0.99–1.00)	∞	0.02 (0.0025–0.12)
**Quantitative SWE parameter**						
*E*_max_ (kPa)	>46.45	83 (70–91) [48/58]	88 (81–93) [123/140]	0.92 (0.88–0.96)	6.82 (4.30–10.80)	0.20 (0.11–0.34)
*E*_mean_ (kPa)	>35.35	83 (70–91) [48/58]	91 (85–95) [128/140]	0.91 (0.86–0.96)	9.66 (5.55–16.80)	0.19 (0.11–0.33)
*E*_ratio_ (kPa)	>4.15	76 (63–86) [44/58]	89 (82–93) [124/140]	0.86 (0.79–0.92)	6.64 (4.09–10.8)	0.27 (0.17–0.43)
*E*_SD_ (kPa)	>10.35	76 (63–86) [44/58]	94 (88–97) [131/140]	0.92 (0.88–0.96)	11.8 (6.17–22.57)	0.26 (0.16–0.41)
Combined quantitative SWE parameters	−0.87	83 (70–91) [48/58]	93 (87–96) [130/140]	0.92 (0.88–0.97)	11.59 (6.30–21.30)	0.18 (0.10–0.33)
BI-RADS category at US	>3	95 (85–99) [55/58]	54 (46–63) [76/140]	0.94 (0.89–0.98)	2.07 (1.71–2.51)	0.09 (0.03–0.29)

Data in parentheses are 95% confidence intervals and data in brackets are raw data. *US*, ultrasonography; *RS*, radiomics signature; *SWE*, shear-wave elastography; *BI-RADS*, Breast Imaging Reporting and Data System; and *AUC*, area under the curve. * *P* values for statistical significance are corrected to 0.008 for multiple testing using Bonferroni correction.

**FIGURE 8:**
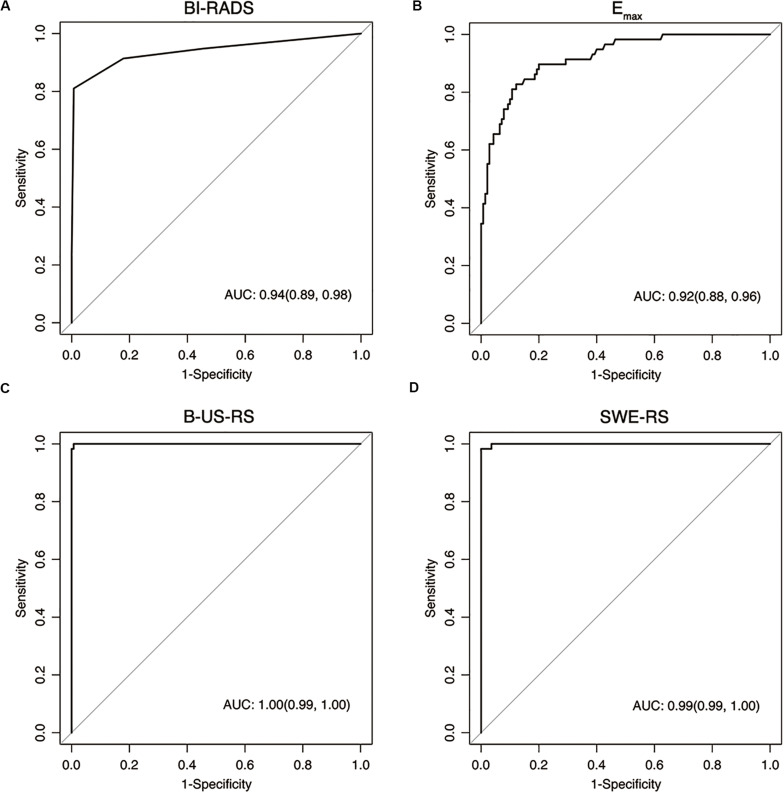
Receiver operating characteristic curves show the diagnostic performance of the Breast Imaging Reporting and Data System (BI-RADS) assessment **(A)**, *E*_max_ **(B)**, deep learning-based radiomics signature of B-mode ultrasonography (B-US-RS; **C**), and deep learning-based radiomics signature of shear-wave elastography (SWE-RS; **D**) in the training cohort. The area under the receiver operating characteristic curves (AUCs) of B-US-RS (AUC = 0.99) and SWE-RS (AUC = 0.99) were both higher than that of *E*_max_ (AUC = 0.92, *P* < 0.001 for both), while there was no significant difference in the AUCs between B-US-RS and SWE-RS (*P* = 0.37), as well as between *E*_max_ and BI-RADS assessment (*P* = 0.36).

The AUCs of B-US-RS and SWE-RS both were 0.99 (95% CI = 0.99–1.00), which were higher than those of *E*_max_ (*P* < 0.001 for both) and BI-RADS assessment (*P* = 0.008 and 0.009, respectively; Figure 8). There was no significant difference in the AUCs between B-US-RS and SWE-RS (*P* = 0.37). The sensitivity and specificity of B-US-RS and SWE-RS were higher than their counterparts of *E*_max_ (*P* = 0.001 and < 0.001, respectively, for B-US-RS; *P* = 0.04 and < 0.001, respectively, for SWE-RS). Among B-US-RS, SWE-RS, *E*_max_, and BI-RADS assessment, only B-US-RS and SWE-RS achieved a positive likelihood ratio greater than 10 and a negative likelihood ratio less than 0.1.

### Diagnostic Performances of B-US-RS, SWE-RS, Quantitative SWE Parameters, and BI-RADS Assessment in the Independent Validation Cohort

The independent validation cohort included 65 masses, of which 46 (70.8%) were benign and 19 (29.2%) were malignant. The diagnostic performances of B-US-RS, SWE-RS, quantitative SWE parameters, and BI-RADS assessment in the validation cohort are shown in Table 4. The quantitative SWE parameters, including *E*_max_, *E*_mean_, *E*_ratio_, and *E*_SD_, were significantly higher in malignant lesions than in benign lesions (*P* < 0.001 for all; Supplementary Figure S1). There were no significant differences in the AUCs among these four quantitative SWE parameters (*P* = 0.22–0.70) and between *E*_max_ (0.93; 95% CI = 0.85–1.00) and the quantitative SWE parameters combined (0.94; 95% CI = 0.88–1.00, *P* = 0.67), as well as between *E*_max_ and BI-RADS assessment (0.99; 95% CI = 0.97–1.00, *P* = 0.18). *E*_max_ had a higher specificity than BI-RADS assessment (*P* < 0.001), and they had similar sensitivity (*P* = 0.07).

**TABLE 4:** Diagnostic performances of B-US-RS, SWE-RS, quantitative SWE parameters, and BI-RADS assessment in the independent validation cohort.

Parameter	Threshold	Sensitivity (%)*	Specificity (%)*	AUC*	Positive likelihood ratio	Negative likelihood ratio
B-US-RS	−1.28	100 (79–100) [19/19]	100 (90–100) [46/46]	1.00 (1.00–1.00)	∞	0
SWE-RS	−0.24	100 (79–100) [19/19]	100 (90–100) [46/46]	1.00 (1.00–1.00)	∞	0
**Quantitative SWE parameter**						
*E*_max_ (kPa)	>46.45	84 (60–96) [16/19]	89 (76–96) [41/46]	0.93 (0.85–1.00)	7.75 (3.31–18.1)	0.18 (0.06–0.50)
*E*_mean_ (kPa)	>35.35	84 (60–96) [16/19]	96 (84–99) [44/46]	0.91 (0.80–1.00)	19.37 (4.92–76.17)	0.16 (0.06–0.47)
*E*_ratio_ (kPa)	>4.15	89 (66–98) [17/19]	87 (73–95) [40/46]	0.90 (0.80–1.00)	6.86 (3.20–14.70)	0.12 (0.03–0.45)
*E*_SD_ (kPa)	>10.35	90 (66–98) [17/19]	96 (84–99) [44/46]	0.95 (0.89–1.00)	20.58 (5.26–80.52)	0.11 (0.03–0.41)
Combined quantitative SWE parameters	−0.87	84 (60–96) [16/19]	96 (84–99) [44/46]	0.94 (0.88–1.00)	19.37 (4.92–76.17)	0.16 (0.06–0.47)
BI-RADS category at US	>3	100 (79–100) [19/19]	54 (39–69) [25/46]	0.99 (0.97–1.00)	2.19 (1.59–3.00)	0 (0–0)

Data in parentheses are 95% confidence intervals and data in brackets are raw data. *US*, ultrasonography; *RS*, radiomics signature; *SWE*, shear-wave elastography; *BI-RADS*, Breast Imaging Reporting and Data System; and *AUC*, area under the curve. * *P* values for statistical significance are corrected to 0.008 for multiple testing using Bonferroni correction.

The AUCs of B-US-RS and SWE-RS both were 1.00 (95% CI = 1.00–1.00). There was no significant difference between B-US-RS and SWE-RS (*P* > 0.99). The AUCs of B-US-RS and SWE-RS were not significantly different from those of *E*_max_ (*P* = 0.12 for both) and BI-RADS assessment (*P* = 0.18 for both; Supplementary Figure S2). The specificities of B-US-RS and SWE-RS were both higher than that of *E*_max_ (*P* = 0.02 for both), while the sensitivity was not significantly different (*P* = 0.07 for both). Among B-US-RS, SWE-RS, *E*_max_, and BI-RADS assessment, only B-US-RS and SWE-RS achieved a positive likelihood ratio greater than 10 and a negative likelihood ratio less than 0.1.

### Diagnostic Performances of B-US-RS, SWE-RS, Quantitative SWE Parameters, and BI-RADS Assessment in the External Validation Cohort

The external validation cohort included 28 masses, of which 18 (64.3%) were benign and 10 (35.7%) were malignant. The diagnostic performances of B-US-RS, SWE-RS, quantitative SWE parameters, and BI-RADS assessment in the validation cohort are shown in Table 5. The quantitative SWE parameters, including *E*_max_ (*P* = 0.001), *E*_mean_ (*P* = 0.002), and *E*_ratio_ (*P* = 0.01), were significantly higher in malignant lesions than in benign lesions, while there was no significant difference between malignant and benign lesions in *E*_SD_ (*P* = 0.28; Supplementary Figure S3). There were no significant differences in the AUCs among these four quantitative SWE parameters (*P* = 0.26–0.96) and between *E*_max_ (0.90; 95% CI = 0.77–1.00) and the quantitative SWE parameters combined (0.88; 95% CI = 0.73–1.00, *P* = 0.72), as well as between *E*_max_ and BI-RADS assessment (0.87; 95% CI = 0.70–1.00, *P* = 0.55). *E*_max_ and BI-RADS assessment had similar specificity (*P* = 0.05) and sensitivity (*P* = 0.26).

**TABLE 5:** Diagnostic performances of B-US-RS, SWE-RS, quantitative SWE parameters, and BI-RADS assessment in the external validation cohort.

Parameter	Threshold	Sensitivity (%)*	Specificity (%)*	AUC*	Positive likelihood ratio	Negative likelihood ratio
B-US-RS	−1.28	100 (79–100) [10/10]	100 (90–100) [18/18]	1.00 (1.00–1.00)	∞	0
SWE-RS	−0.24	100 (79–100) [10/10]	100 (90–100) [18/18]	1.00 (1.00,1.00)	∞	0
**Quantitative SWE parameter**						
*E*_max_ (kPa)	>46.45	70 (35–92) [7/10]	89 (64–98) [16/18]	0.90 (0.77–1.00)	6.30 (1.60–24.75)	0.34 (0.13–0.88)
*E*_mean_ (kPa)	>35.35	70 (35–92) [7/10]	94 (71–100) [17/18]	0.86 (0.69–1.00)	12.6 (1.80–88.34)	0.32 (0.12–0.82)
*E*_ratio_ (kPa)	>4.15	70 (35–92) [7/10]	89 (64–98) [16/18]	0.86 (0.69–1.00)	6.30 (1.60–24.75)	0.34 (0.13–0.88)
*E*_SD_ (kPa)	>10.35	80 (44–96) [8/10]	89 (64–98) [16/18]	0.89 (0.75–1.00)	7.20 (1.88–27.58)	0.22 (0.06–0.79)
Combined quantitative SWE parameters	−0.87	70 (35–92) [7/10]	94 (71–100) [17/18]	0.88 (0.73–1.00)	12.6 (1.80–88.34)	0.32 (0.12–0.82)
BI-RADS category at US	>3	90 (54–100) [9/10]	39 (18–64) [7/18]	0.87 (0.70–1.00)	1.47 (0.96–2.25)	0.26 (0.03–1.96)

Data in parentheses are 95% confidence intervals and data in brackets are raw data. *US*, ultrasonography; *RS*, radiomics signature; *SWE*, shear-wave elastography; *BI-RADS*, Breast Imaging Reporting and Data System; and *AUC*, area under the curve. * *P* values for statistical significance are corrected to 0.008 for multiple testing using Bonferroni correction.

The AUCs of B-US-RS and SWE-RS both were 1.00 (95% CI = 1.00–1.00). There was no significant difference between B-US-RS and SWE-RS (*P* > 0.99). The AUCs of B-US-RS and SWE-RS were not significantly different from those of *E*_max_ (*P* = 0.13 for both) and BI-RADS assessment (*P* = 0.14 for both; Supplementary Figure S4). The specificity and sensitivity of B-US-RS and SWE-RS were similar to those of *E*_max_ (*P* = 0.13 for both and *P* = 0.06 for both, respectively). Among B-US-RS, SWE-RS, *E*_max_, and BI-RADS assessment, only B-US-RS and SWE-RS achieved a positive likelihood ratio greater than 10 and a negative likelihood ratio less than 0.1.

## Discussion

Our study showed that the deep learning-based radiomics signatures developed either from the B-mode US or the SWE images had a robust and superior diagnostic performance in classifying breast masses. The specificities of both were higher than those of the quantitative SWE parameters and BI-RADS assessment.

B-mode US and SWE are frequently used in the workup of patients with breast lesions. The classification of breast lesions on the B-mode US is primarily based on the morphological features with a resultant BI-RADS category. This approach has high sensitivities ranging from 95 to 97%, but low specificities ranging from 55 to 68%, in the differentiation between benign and malignant breast masses (33). Quantitative SWE parameters have been reported to be able to classify breast lesions with a specificity of 86% and a sensitivity of 84% (14). Among the quantitative SWE parameters, *E*_max_, *E*_mean_, *E*_ratio_, and *E*_SD_ are the most commonly used indexes for the differential diagnosis (7, 22). It has been demonstrated that quantitative SWE measurement, such as *E*_max_, has better diagnostic performance than radiologist interpretation of BI-RADS on B-mode US in differentiating malignant breast tumors from benign ones (7, 13). In our study, among the four quantitative SWE parameters, *E*_max_ had the highest AUC in the training cohort and was the best-performing quantitative SWE parameter in classifying breast lesions. The specificity of *E*_max_ was higher than that of the BI-RADS assessment both in the training cohort and in the independent validation cohort, which was in agreement with other studies (7, 13). Moreover, our results showed that the combination of all four quantitative SWE parameters did not achieve better performance than *E*_max_ either in the training cohort or validation cohort, which was consistent with the finding of the BE1 Multinational Study (7). Taken together, the diagnostic performances of *E*_max_ and BI-RADS assessment are comparable, and the addition of *E*_max_ can improve the specificity without loss of sensitivity for classifying breast lesions.

Recently, radiomics analysis based on US images has been shown to be able to improve the diagnostic accuracy for breast tumor classification with an AUC up to 0.922 (34). A convolutional neural network-based radiomics model has been proposed to automatically extract features from SWE data for classifying malignant and benign breast tumors, reaching an accuracy of 95.8%, a sensitivity of 96.2%, and a specificity of 95.7% (35). Besides, a deep learning model has been developed to automatically extract features from the SWE image and classify breast tumors, reaching an accuracy of 93.4%, a sensitivity of 88.6%, a specificity of 97.1%, and an AUC of 0.947 (36). However, in these two deep learning studies, the B-mode US data were not used for analysis. The performances of two deep learning models were not compared with the quantitative SWE parameters and BI-RADS assessment. In our study, deep learning-based radiomics analysis was applied to SWE images as well as B-mode US images. The radiomics signatures developed from B-mode US and the SWE images showed comparable, superior performance for the classification of breast masses in the training (AUC = 0.99 for both), independent validation (AUC = 1.00 for both), and external validation cohorts (AUC = 1.00 for both). Comparatively, the SWE-RS in our study had a higher diagnostic performance than that reported previously (35, 36). Moreover, our study showed that the diagnostic performances of B-US-RS and SWE-RS were both higher than those of the quantitative SWE parameters and BI-RADS assessment. These results suggest that either the SWE-based or B-mode US-based radiomics signature with a deep learning approach can be applied to further improve the classification ability for breast masses. Based on these radiomics signatures, a patient with a malignant breast tumor would be correctly selected for prompt interventional procedure, while a patient with a benign breast tumor would safely receive follow-up or continued surveillance rather than an invasive biopsy. The integration of deep learning-based radiomics signatures into B-mode US or SWE would be favorable for clinical decision making in patients with breast masses.

Our study has several limitations. Firstly, this study was a bicenter study, and images were obtained on the equipment from the same vender. Considering the superior performance, these radiomics signatures are worthy of further validation in future large-scale, multicenter, and multi-vendor studies. Secondly, only the two-dimensional, but not three-dimensional, SWE images were used for deep learning-based radiomic feature extraction and radiomics signature development. The three-dimensional SWE was not readily available in clinics, and it was considered that the diagnostic performance of the three-dimensional SWE image is similar to that of the two-dimensional SWE or even inferior to the two-dimensional SWE after adding to B-mode US (37, 38). Thirdly, image segmentation of breast lesions was performed manually in our study, which was a time-consuming task for a large database. Future automatic segmentation methods could be expected with the development of a deep learning-based radiomic feature extraction algorithm.

In conclusion, two robust deep learning-based radiomics signatures developed from B-mode US images and SWE images have been described. Both of them outperformed the quantitative SWE parameters and BI-RADS assessment for classifying breast masses. The integration of this deep learning-based radiomics approach to B-mode US and SWE would help improve the classification ability of the US for breast lesions.

## Data Availability Statement

The raw data supporting the conclusions of this article will be made available by the authors, without undue reservation.

## Ethics Statement

The studies involving human participants were reviewed and approved by Ethics Committees of Sun Yat-sen Memorial Hospital, Sun Yat-sen University, and Guangdong Provincial Traditional Chinese Medicine Hospital. The Ethics Committee waived the requirement of written informed consent for participation.

## Author Contributions

XZ, ML, and CZ conceived and designed the study. ML, JW, and BO collected the clinical and image data and performed image pre-processing. ZY, HL, and XW analyzed the image data and performed the statistical analysis. XZ and ML wrote the manuscript. JS and BL reviewed and edited the manuscript. All authors approved the final manuscript.

## Conflict of Interest

The authors declare that the research was conducted in the absence of any commercial or financial relationships that could be construed as a potential conflict of interest.
