# Supplementary figures and images for: Deep Learning-Based Radiomics of B-Mode Ultrasonography and Shear-Wave Elastography: Improved Performance in Breast Mass Classification
Source: Front Oncol. 2020 Aug 28;10:1621. doi: 10.3389/fonc.2020.01621 (PMC7485397; doi:10.3389/fonc.2020.01621)

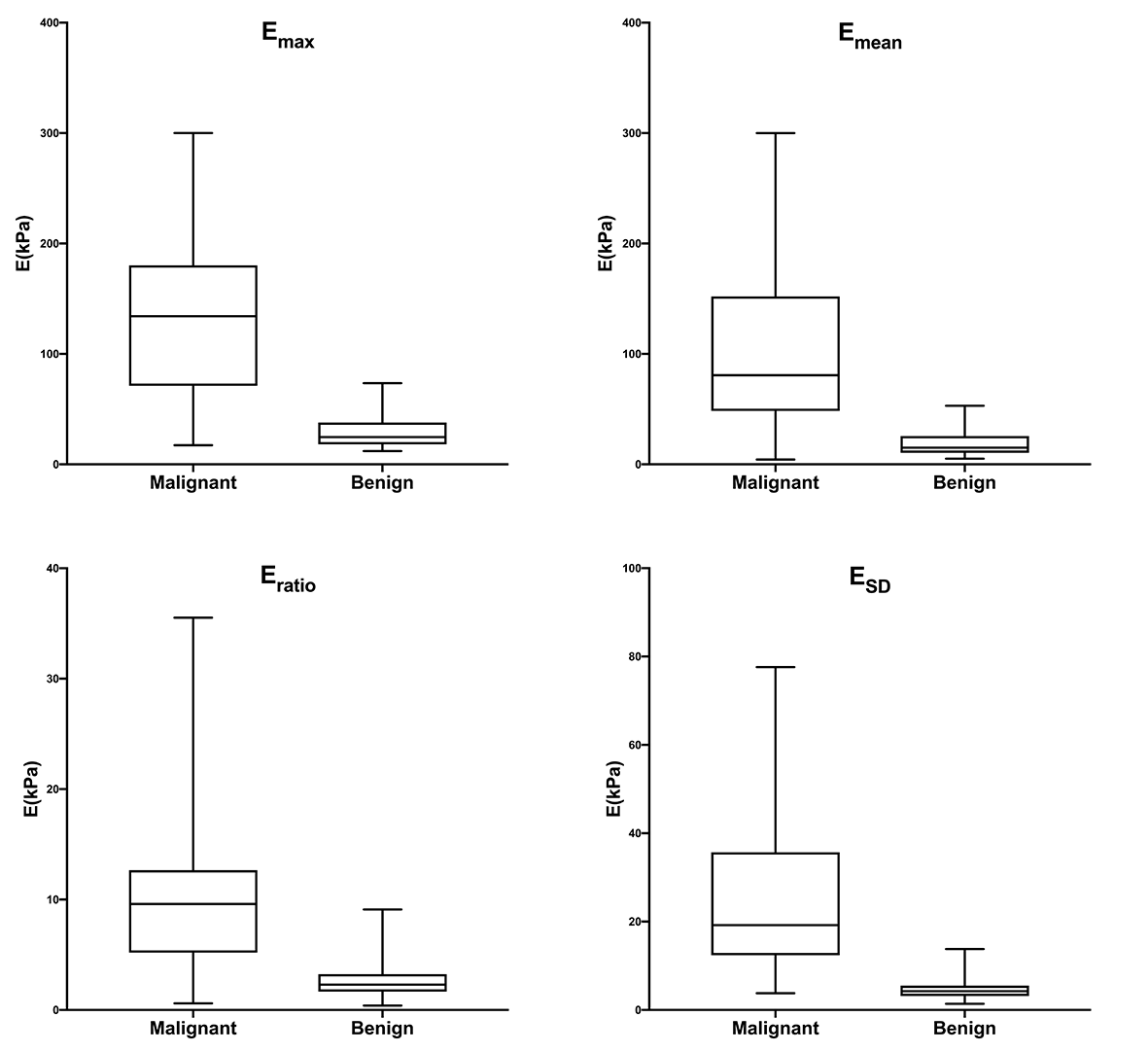

Supplement: Supplementary file 1 [file Image_1.TIF]

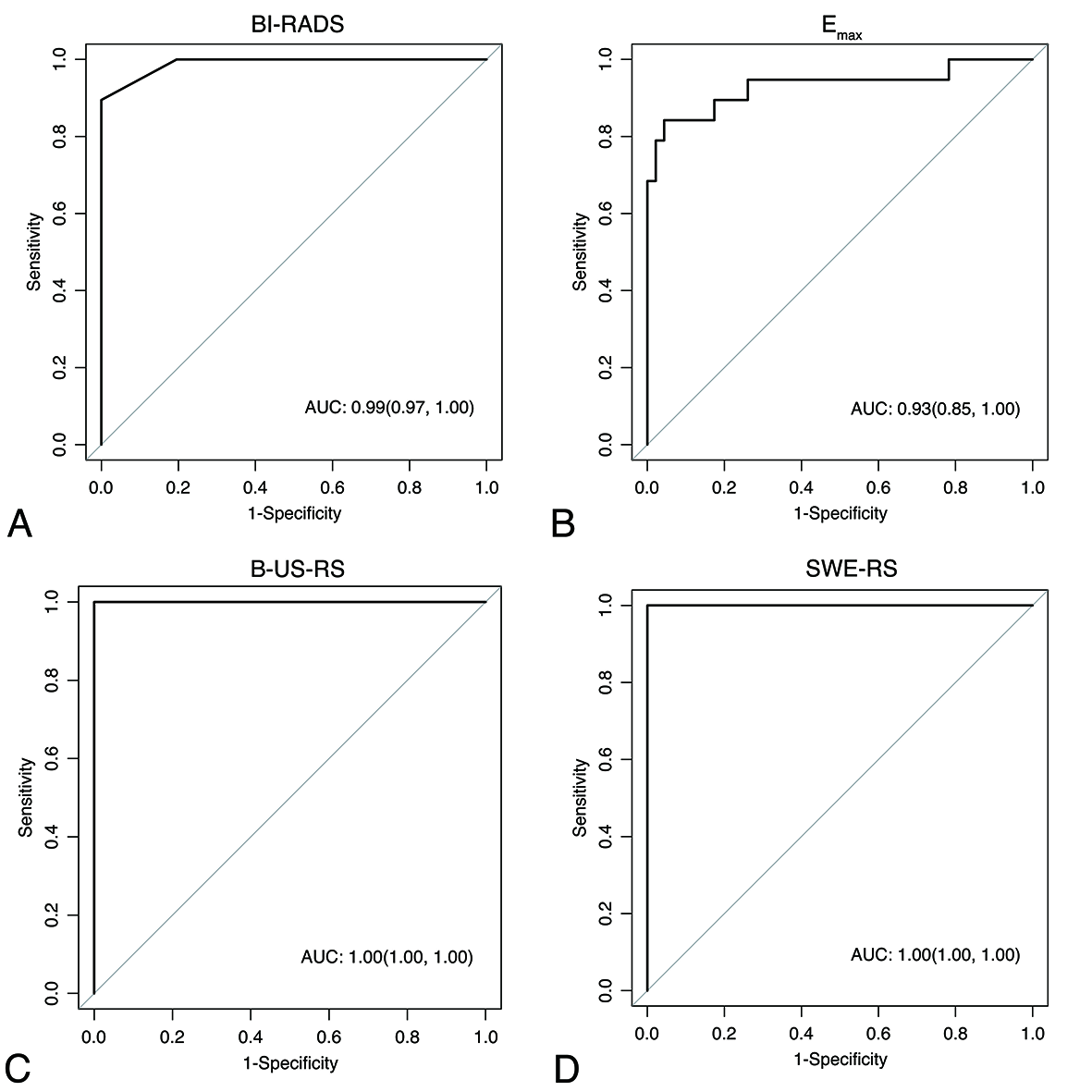

Supplement: Supplementary file 2 [file Image_2.TIF]

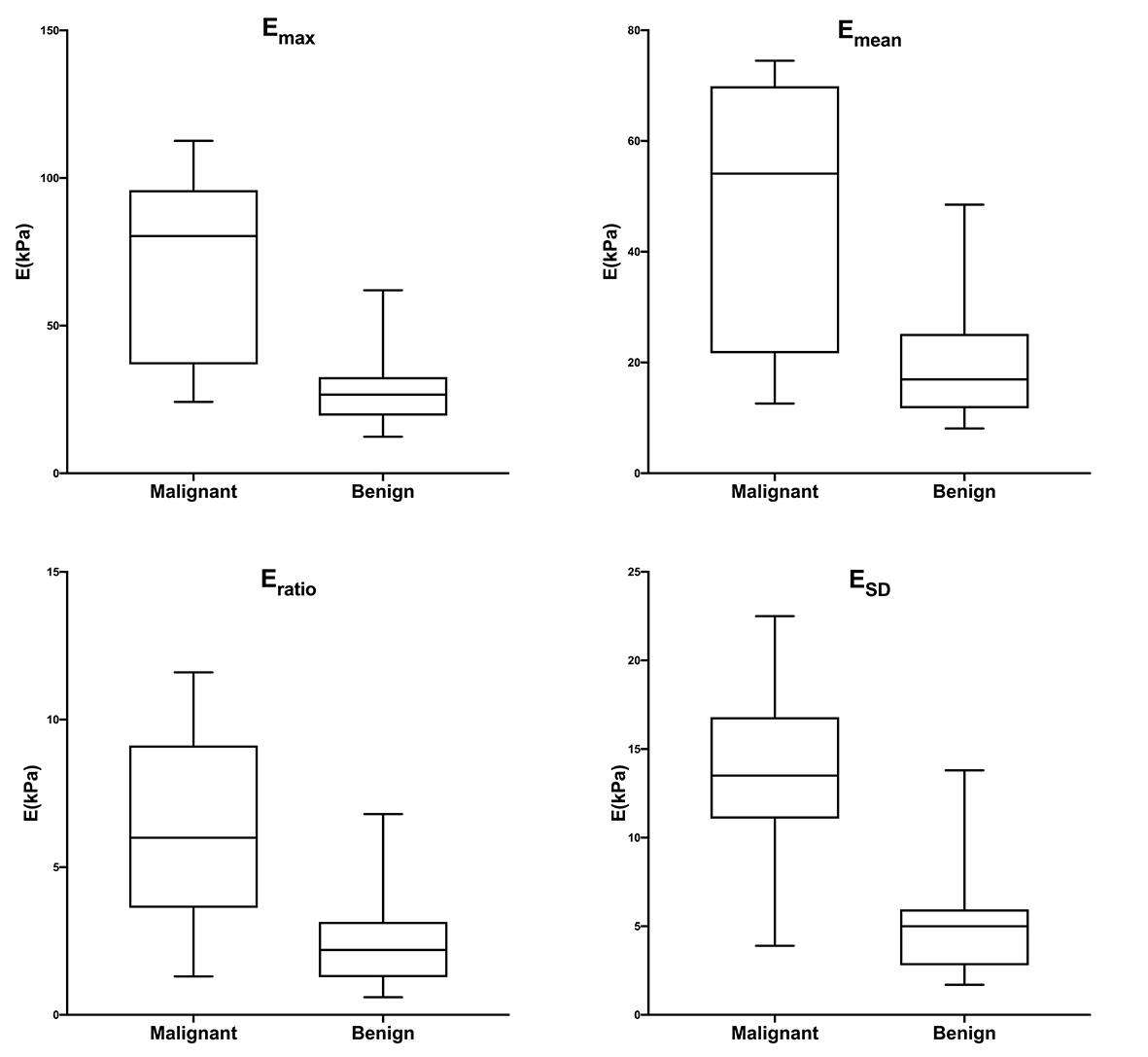

Supplement: Supplementary file 3 [file Image_3.TIF]

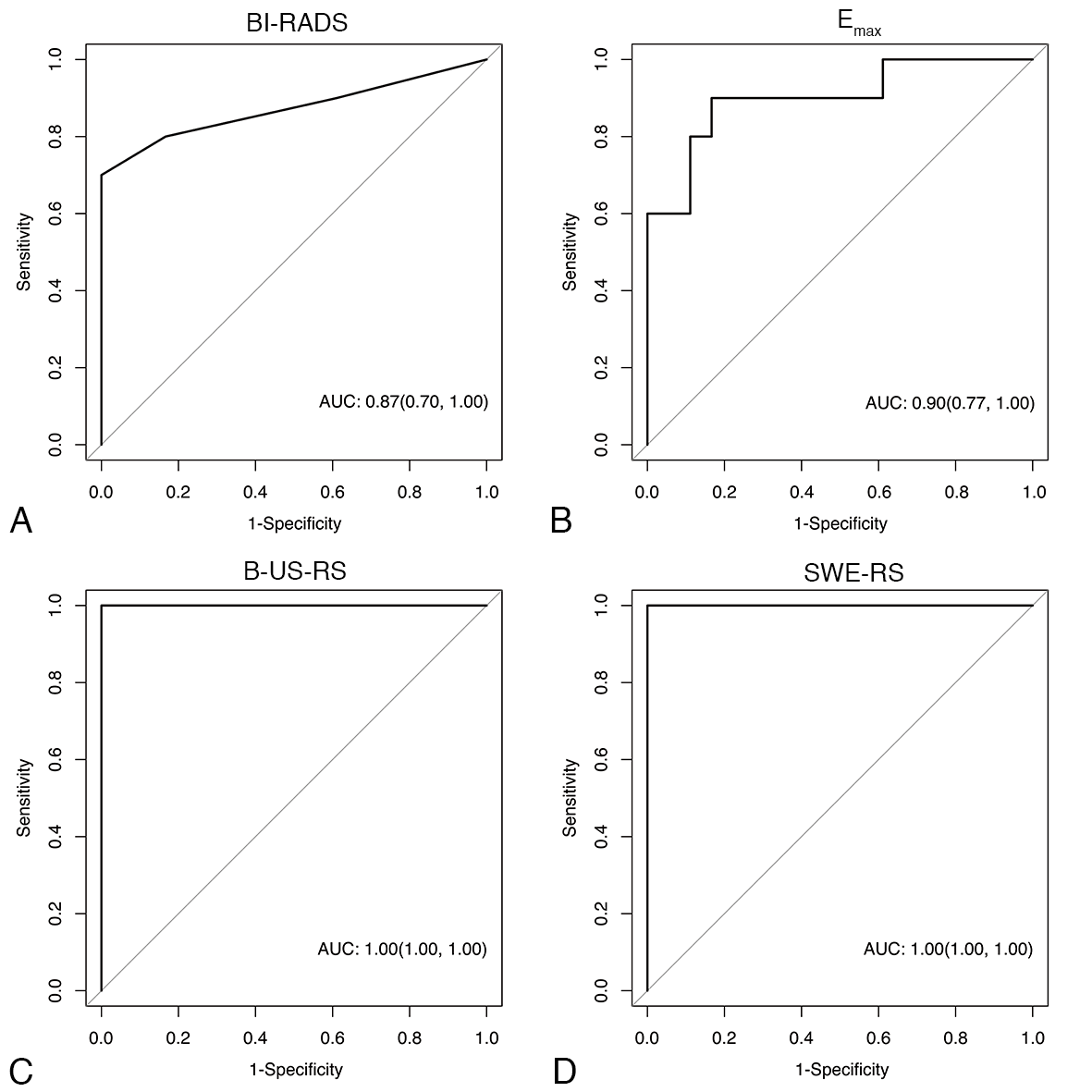

Supplement: Supplementary file 4 [file Image_4.TIF]
