# Supplementary material for: Deep Learning-Based Radiomics of B-Mode Ultrasonography and Shear-Wave Elastography: Improved Performance in Breast Mass Classification
Source: Front Oncol. 2020 Aug 28;10:1621. doi: 10.3389/fonc.2020.01621 (PMC7485397; doi:10.3389/fonc.2020.01621)
Supplement: Supplementary file 5 [file Table_1.DOCX]

| **Examination** | **Radiomics Score** | **Median (25th percentile, 75th percentile) of Radiomics Score** |
| --- | --- | --- |
| US | Malignant Lesion in Training Cohort | 3.60 (3.44, 3.60) |
| US | Benign Lesion in Training Cohort | -4.59 (-5.46, -3.75) |
| US | Malignant Lesion in Independent Validation Cohort | 3.60 (3.56, 3.60) |
| US | Benign Lesion in Independent Validation Cohort | -4.58 (-5.11, -3.90) |
| US | Malignant Lesion in External Validation Cohort | 3.09 (1.92, 3.55) |
| US | Benign Lesion in External Validation Cohort | -4.49 (-5.27, -3.95) |
| SWE | Malignant Lesion in Training Cohort | 2.50 (2.50, 2.50) |
| SWE | Benign Lesion in Training Cohort | -3.63 (-4.13, -2.95) |
| SWE | Malignant Lesion in Independent Validation Cohort | 2.50 (2.50, 2.50) |
| SWE | Benign Lesion in Independent Validation Cohort | -3.49 (-3.94, -3.10) |
| SWE | Malignant Lesion in External Validation Cohort | 2.34 (1.33, 2.50) |
| SWE | Benign Lesion in External Validation Cohort | -3.48 (-4.05, -3.14) |

**Table S1. Radiomics Scores of Benign and Malignant Lesions in the Training, Independent Validation, and External Validation Cohorts**

Note.— US = ultrasonography, SWE = shear-wave elastography.

**Supplementary Figure Legends**

**Figure S1.** Box-and-whisker plots of E_max_, E_mean_, E_ratio_, and E_SD_ in malignant and benign lesions in the independent validation cohort. The top and bottom of each box represent 75th and 25th percentiles, respectively. The horizontal line in each box represents the median, and the top and bottom of the whiskers represent the minimum and maximum value, respectively. E_max_, E_mean_, E_ratio_, and E_SD_ were significantly higher in malignant lesions than in benign lesions (*P* < 0.001 for all).

**Figure S2.** Receiver operating characteristic curves show the diagnostic performance of Breast Imaging Reporting and Data System (BI-RADS) assessment (A), E_max_ (B), deep learning-based B-mode US radiomics signature (B-US-RS) (C), and deep learning-based shear-wave elastography radiomics signature (SWE-RS) (D) in the independent validation cohort. The area under the receiver operating characteristic curves (AUCs) of B-US-RS (AUC = 1.00) and SWE-RS (AUC = 1.00) had no significant difference compared with that of E_max_ (AUC = 0.93, *P* = 0.12 for both) and BI-RADS assessment (AUC = 0.99, *P* = 0.18 for both). There was no significant difference in AUCs between B-US-RS and SWE-RS (*P* > 0.99).

**Figure S3.** Box-and-whisker plots of E_max_, E_mean_, E_ratio_, and E_SD_ in malignant and benign lesions in the independent validation cohort. The top and bottom of each box represent 75th and 25th percentiles, respectively. The horizontal line in each box represents the median; and the top and bottom of the whiskers represent the minimum and maximum value, respectively. E_max_ (*P* = 0.001), E_mean_ (*P* = 0.002), and E_ratio_ (*P* = 0.01) were significantly higher in malignant lesions than in benign lesions (*P* < 0.001 for all), while no significantly difference was found between malignant and benign lesions in E_SD_ (*P* = 0.28).

**Figure S4.** Receiver operating characteristic curves show the diagnostic performance of Breast Imaging Reporting and Data System (BI-RADS) assessment (A), E_max_ (B), deep learning-based B-mode US radiomics signature (B-US-RS) (C), and deep learning-based shear-wave elastography radiomics signature (SWE-RS) (D) in the external validation cohort. The area under the receiver operating characteristic curves (AUCs) of B-US-RS (AUC = 1.00) and SWE-RS (AUC = 1.00) had no significant difference compared with that of E_max_ (AUC = 0.90, *P* = 0.13 for both) and BI-RADS assessment (AUC = 0.87, *P* = 0.14 for both). There was no significant difference in AUCs between B-US-RS and SWE-RS (*P* > 0.99).
